# Ethnic Differences in the Quality of the Interview Process and Implications for Survey Analysis: The Case of Indigenous Australians

**DOI:** 10.1371/journal.pone.0130994

**Published:** 2015-06-19

**Authors:** Francisco Perales, Bernard Baffour, Francis Mitrou

**Affiliations:** 1 ARC Centre of Excellence for Children and Families over the Life Course, Institute for Social Science Research, The University of Queensland, Postal address: ISSR, Building 39a (GPN3), The University of Queensland, QLD 4072, Brisbane, Australia; 2 ARC Centre of Excellence for Children and Families over the Life Course, Telethon Kids Institute, The University of Western Australia, Postal address: Telethon Kids Institute, 100 Roberts Road, Subiaco, WA 6008, Perth, Australia; Queensland University of Technology, AUSTRALIA

## Abstract

Comparable survey data on Indigenous and non-Indigenous Australians are highly sought after by policymakers to inform policies aimed at closing ethnic socio-economic gaps. However, collection of such data is compromised by group differences in socio-economic status and cultural norms. We use data from the Household, Income and Labour Dynamics in Australia Survey and multiple-membership multilevel regression models that allow for individual and interviewer effects to examine differences between Indigenous and non-Indigenous Australians in approximate measures of the quality of the interview process. We find that there are both direct and indirect ethnic effects on different dimensions of interview process quality, with Indigenous Australians faring worse than non-Indigenous Australians in all outcomes *ceteris paribus* . This indicates that nationwide surveys must feature interview protocols that are sensitive to the needs and culture of Indigenous respondents to improve the quality of the survey information gathered from this subpopulation.

## Introduction

The amount of cross-sectional and longitudinal Australian survey data available to researchers and policymakers for social commentary and decision-making has grown exponentially in the past two decades, paralleling the rise of computing power to simplify data collection, enable large data storage, and speed up statistical data analysis. As a consequence, more information is being collected about more of the population in Australia. This includes the general population and the various cultural, geographical, and socio-economic population subgroups that comprise Australian society.

One of the subpopulations of most interest in Australia are persons from an Aboriginal and/or Torres Strait Islander background (referred hereafter as Indigenous Australians), comprising individuals who identify as descendants of the original inhabitants of the land prior to colonization [1]. Around 550,000 Australians identified as Indigenous in the 2011 Australian Census of Population and Housing, compared to around 19,900,000 Australians who identified as non-Indigenous [2]. Thus, Indigenous people comprise about 3% of the total population in Australia. Information about Indigenous Australians is highly sought after by policy makers to underpin decisions about policies and programs developed specifically for this sub-population. Indigenous Australians are amongst the most disadvantaged population groups in Australia, suffering ongoing effects from European colonization. In its earlier phase, these included human-rights abuses and institutionalized discrimination in the form of genocide, land appropriation and removal of children from their families. Even in more socially enlightened times, the stubbornly persistent gap in socio-economic outcomes between Indigenous and non-Indigenous Australians has remained an important social issue for Australian society and a focus of policy planning. From the 1970s successive Australian Governments have supported policies designed to help rectify the negative impacts on Indigenous wellbeing of policies from previous eras. Early policies focused on achieving ‘statistical equality’ and more recent ones changed the discourse to one of ‘practical reconciliation’ [3]. Most recently, the *Closing the Gap* initiative brings together State, Territory and Commonwealth Governments to address Indigenous disadvantage [4].

Indicators derived from social survey data are routinely used by governments to monitor the progress of these initiatives, and so comparable good-quality data on both Indigenous and non-Indigenous Australians is of utmost importance to understand the social determinants of ethnic-based socio-economic gaps in outcomes in Australia. However, collecting such data in a reliable way is challenging, with the 2008 National Indigenous Reform Agreement (NIRA) specifically emphasizing the need to improve data quality on Indigenous Australians as a means of closing the outcome gap. There is a long-running debate on the inherent trade-off between survey practice standardization to achieve consistency and the appropriateness of survey tools to the Indigenous cultural context [5]. As will be discussed further later in the paper, Indigenous Australians are a largely distinctive sub-population in Australia. Not only do they have, on average, lower socio-economic status and live in more remote and deprived areas, but also hold cultural values and worldviews that differ from those of non-Indigenous Australians. As a result, Indigenous Australians have below average propensities to agree to participate in social surveys, and a higher propensity to drop out of longitudinal surveys that follow individuals over time. We argue that, additional to these, because population surveys are designed to fit the circumstances and cultural norms of the non-Indigenous majority, Indigenous Australians may have a lower predisposition and ability to complete the survey in the intended, optimal manner. Both material circumstances and Indigenous culture are expected to play a role in the emergence of ethnic differences in the quality of the interview process (QotIP). Any such differences would have important practical and policy implications, as they would be suggestive that surveys designed for administration in the general population provide information of different quality for Indigenous and non-Indigenous Australians. This would in turn affect the reliability of ethnic comparisons made using those data sources and question the applicability of any associated research findings for policy purposes.

In this paper we aim at establishing whether there are differences in QotIP between Indigenous and non-Indigenous Australian survey respondents, and whether any effects are a direct product of cultural disparities or indirect and mediated by factors such as family demographics, human or economic capital and interview conditions. We do so by exploiting the properties of a powerful dataset, the Household, Income and Labour Dynamics in Australia (HILDA) Survey to estimate state-of-the-art multiple-membership three-level regression models. Key findings indicate that there are both direct and indirect ethnic effects on different dimensions of interview process quality, with Indigenous Australians faring worse than non-Indigenous Australians in all outcomes *ceteris paribus*. We read these as indicating that nationwide surveys must feature interview protocols that are sensitive to the needs and culture of Indigenous respondents to improve the quality of the survey information gathered from this subpopulation

## Background

### The quality of the interview process

From a *total survey error* perspective, survey data errors may emerge from four sources: sampling, coverage, non-response, and measurement error [6]. Here, we are primarily concerned about the last of the four sources of error: measurement error. Specifically, we focus on the potential for measurement error to arise due a suboptimal interview process. We define the ‘interview process’ as the compendium of verbal and non-verbal interactions between the interviewer and the interviewee during the administration of a face-to-face structured survey questionnaire, and start from the premise that such a process can have different degrees of “quality”.

The quality of the interview process (QotIP) is determined by how conducive the interview process is to gathering high-quality information from the respondent. QotIP will be high when the interaction between interviewer and interviewee is characterized by trust, mutual understanding and mutual cooperation. Conversely, QotIP will be low when the opposite holds true. Additionally, other external factors can also affect QotIP. For instance, most survey designs expect and promote that the interview takes place in an indoors setting, with just the respondent and the interviewer in the room. When others are present, interviewees are not expected to have their responses influenced. Deviations from this pattern are considered undesirable and can result in information of suboptimal quality being gathered, with empirical evidence suggesting that aspects of QotIP such as respondent cooperation, enjoyment and comprehension of the survey questions and goals positively affect respondent retention rates [6–8].

QotIP is thus a complex and multidimensional concept, and poor QotIP can emerge from several sources, including bad interviewer practices, unknowledgeable or suspicious respondents, and interview environments which clash with rigid interview protocols (e.g. overcrowded houses). We examine four complementary proxy measures of QotIP (influenced responses, suspicious respondents, comprehension issues, and uncooperative respondents), which will be explained in more detail in the methods section.

There is ample international evidence on how minority status affects survey coverage and survey non-response, as well as attrition from panel studies [9–11]. However, little previous research has examined the correlates of QotIP, and none of it in the Australian context. So far, most of the academic interest surrounding QotIP measures has been on how they are affected by survey features—e.g. telephone vs. face-to-face interviews, as in Holbrook et al. [12]—rather than personal characteristics, or how they affect some other survey outcome—e.g. consent to link administrative to survey data, as in [13] or panel attrition [7].

### The role of Indigeneity: Direct and indirect effects

Indigenous cultural imperatives may result in understandings of survey questions and response categories that can be different from other sectors of the Australian community, or involve norms and rituals concerning social interactions that diverge from standardized survey protocols. A key goal of this paper is to tease out the independent contributions to QotIP of (i) Indigenous cultural traditions and worldviews (which we define as *direct* effects) and (ii) socio-demographic and economic traits that may be disproportionately present in the Indigenous Australian subpopulation (which we define as *indirect* effects).

#### Indirect effects: socio-demographic, socio-economic, and geographical differences

Since colonial times Indigenous Australians have experienced high rates of socio-economic deprivation, poverty and social exclusion. Despite steady improvement in Indigenous Australian socio-economic outcomes since the 1970s [3], they still fare substantially worse than non-Indigenous Australians in human capability resource endowments such as education, employment, income and health [14–16] and tend to live in more deprived areas than non-Indigenous Australians [17–18]. As a result, Indigenous Australians experience substantially higher prevalence of ‘deep and persistent exclusion’ (11%) than non-Indigenous Australians (4%) [19]. While these socio-economic outcomes are determined by ‘normative criteria’ and may or may not be important to different Indigenous peoples [1, 20], they are likely related to poor QotIP. As Sims notes *“for most Aboriginal people*, *day-to-day existence is a difficult struggle to meet sets of basic needs”* and so *“providing information is given a much lower priority than attempts to make basic family and community needs”* ([21], page 52). Given this, it is likely that any associations between Indigenous ethnic background and QotIP might be indirect and product of the uneven distribution of socioeconomic traits across the Indigenous and non-Indigenous subpopulations.

Economic and sociological evidence also points towards divergences by ethnic background in other factors known or suspected to influence QotIP. For example, Indigenous Australians are much more likely than their non-Indigenous counterparts to live in overcrowded households [22] and in households with (many) children [23], which may lower QotIP via social desirability biases, influenced responses and interview situations that are conducive to cognitive errors on the part of respondents. Additionally, many Indigenous Australians speak English as a second language [24]. Others use language registers that are different to those employed by the non-Indigenous population [25], which may impede understanding of survey questions and interviewer prompts. Additionally, the disproportionate rates of geographical mobility amongst Indigenous Australians might result in shorter stays in panel studies and a higher prevalence of change in their interviewers [20], both of which are associated with poor QotIP [7]. Furthermore, the relatively high fertility and mortality rates of Indigenous Australians mean that on average Indigenous Australians are younger than non-Indigenous Australians [26], and thus more of them fall within age groups for which QotIP is generally poor.

#### Direct effects: indigenous cultural protocols

In addition to ethnic differences in QotIP that run through the ‘indirect’ channels outlined in the previous section, there may also be more ‘direct’ effects due to cultural differences between Indigenous and non-Indigenous Australians. Despite the diversity of cultures and circumstances of different Australian Indigenous peoples, they share important commonalities that shape their identities and set them apart from non-Indigenous Australians [5]. Indigenous Australian culture is tradition-oriented as well as kinship- and community-based, and often features complex ethics of reciprocity within a lively informal economy, fuzzy household structures and significant extra-household networks. Indigenous Australians display higher than average rates of geographic mobility and remote-area residence, with elements of hunter-gatherer lifestyles still being prevalent in many remote communities [5, 27–28].

Indigenous peoples’ values and social relations have been shown to affect their participation in and response to surveys in the past [20], with long-running evidence of difficulties in securing the trust of Indigenous interviewees, particularly when sensitive information is asked [29]. The sources of such distrust amongst Indigenous Australians have been traced to a long history of discrimination and exploitation since colonial times, a lack of knowledge on the purpose of data collection, and a lack of relevance of the survey questions to their circumstances and a resulting perception of the whole exercise being ‘pointless’ [5]. Relatedly, because Indigenous conversational protocols stress meaning and typically feature longer interactions, Indigenous Australian respondents often feel ‘alienated’ or frustrated by rigidly structured face-to-face surveys that do not enable them to appropriately describe their complex life circumstances [21].

Hunter and Smith also highlight how in smaller surveys of Indigenous people and communities culturally relevant factors such as communication styles and communication patterns influenced the conduct of research [20]. For instance, they describe how interviews were generally conducted *“in public areas*, *invariably with numerous children present”*, with many turning into *“impromptu ‘focus groups’”* ([20], page 264). They also report a tendency amongst some Indigenous people to *“say yes when you mean no”* as well as a desire to *“avoid giving bad news”* ([20], page 266). It has also been argued that many Indigenous Australians may feel that they talk on behalf of their communities rather than as individuals when approached by interviewers [21].

### Existing empirical evidence

The specific case of Indigenous Australians has received virtually no attention in the empirical quantitative literature on QotIP, despite the policy relevance of this population subgroup. There is, however, strong evidence of differences in total survey error for estimates pertaining the Indigenous and non-Indigenous Australian populations.

First, Indigenous Australians are amongst the most ‘hard-to-reach’ population groups in Australia and often have characteristics that make them ‘out of scope’ for most population surveys [30]. For example, by 2011 5% of Indigenous Australians but just 0.35% of non-Indigenous Australians were homeless [31]. Similarly, age-standardized imprisonment rates in 2013 were 15 times higher for Indigenous (1,959 per 100,000 adults) than non-Indigenous Australians (131 per 100,000 adults) [32]. Additionally, ‘remote’ and/or ‘very remote’ areas that contain relatively large shares of Indigenous Australians are out of scope for many population surveys, such as the Longitudinal Study of Australian Children (LSAC), the HILDA Survey and the General Social Survey (GSS) series. The 2011/2013 Australian Health Survey has gone as far as to explicitly exclude Indigenous communities due to insufficient time for community consultation [33]. There are also well-known issues in the coverage of Indigenous Australians in official statistics. For instance, the net undercount of Indigenous Australians in the 2006 Australian Census of population and Housing was 17%, compared to just 6% for non-Indigenous Australians [34].

Second, there is evidence that Indigenous Australians are more likely to decline to participate in population surveys. This is true for many of the recent, major social surveys in Australia. For example, the General Social Survey conducted from August to November 2010 had a response rate of 88% [35], whereas the Indigenous version of the survey, the National Aboriginal and Torres Strait Islander Social Survey, conducted from August 2008 to April 2009 had an 83% response rate [36].

Third, Indigenous Australians are also more likely to stop participating in longitudinal surveys. For instance, in LSAC (beginning in 2004) 80% of the full sample was retained by 2012 (Wave 5), compared to just 60% of the subsample of Indigenous families [37]). Similarly, in the HILDA Survey, 57% of non-Indigenous respondents were successfully re-interviewed in all 12 survey waves, relative to just 43.6% of respondents from an Indigenous background [38].

Given this evidence, it is likely that there are also divergences in QotIP between Indigenous and non-Indigenous Australians. However, to our knowledge, not a single study has systematically examined these. This is alarming, given the importance of ethnic comparisons for academic and policy purposes stressed before. This is the gap that this paper intends to fill.

### Research hypotheses

Based on the theoretical and empirical literature discussed so far, we elaborate several simple, testable hypotheses on the associations between Indigenous ethnic background and QotIP. First, we expect that:

*Hypothesis 1. The interview process for Indigenous Australians will be of poorer quality than for non-Indigenous Australians.*

This can be tested by comparing the outcomes of Indigenous and non-Indigenous respondents using descriptive statistics and regression models with no predictors other than Indigenous status. If Hypothesis 1 holds, then it becomes important to explore the mechanisms that may account for the ethnic discrepancies in QotIP, particularly whether these have a cultural basis. From this we develop two competing hypotheses:

*Hypothesis 2a. Lower interview process quality amongst the Indigenous subpopulation will run through a direct channel due to cultural differences across ethnic groups.*

*Hypothesis 2b. Lower interview process quality amongst the Indigenous subpopulation will run through an indirect channel via group differences in socio-demographic traits, human and economic capital and interview conditions.*

The defining piece of evidence to determine which of these two counter hypotheses holds in these data is the statistical significance of the predicted effect of Indigenous status on interview process outcomes in fully specified models that account for potentially mediating factors. If Indigeneity remains a significant predictor of QotIP in fully specified models, then Hypothesis 2a would be supported, with evidence of a *direct* ethnic effect. Alternatively, if the predicted impact of Indigeneity on QotIP becomes statistically insignificant in the fully specified models, then Hypothesis 2b would be supported, with evidence of an *indirect* ethnic effect.

The following section introduces the data and methods that we use to test these hypotheses.

## Data and Methods

Our aim is to establish whether associations exist between respondents’ Indigenous ethnic background and the quality of the information gathered in social surveys of the general population. A full examination of this requires the use of a data source that incorporates several features that are relatively rare in isolation, and extremely rare in combination. These include (i) a face-to-face interview setting; (ii) a sufficiently large subsample of individuals from an Indigenous background for robust analysis, (iii) data on QotIP that is available to the researcher, (iv) means to identify interviewers to account for unobserved interviewer effects, (v) repeated observations from the same individuals over time to account for unobserved individual effects, and (vi) an encompassing set of observable socio-demographic control variables on factors that may confound the associations of interest. Fortunately, the HILDA Survey fulfils all of these criteria in a way that no other dataset that we know of does. The HILDA Survey is a large-scale household-based multipurpose panel study that is largely representative of the Australian population in 2001 and that collects annual information from the same respondents between 2001 and 2012 [39]).

The HILDA Survey contains information on ethnic background collected when respondents are first interviewed via a survey question that reads: *“Are you of Aboriginal or Torres Strait Islander origin*?*”*. Respondents can choose between the categories ‘Not of Indigenous origin’, ‘Aboriginal’, ‘Torres Strait Islander’ and ‘Both Aboriginal and Torres Strait Islander’. For the purpose of this research, we combine categories ‘Aboriginal’, ‘Torres Strait Islander’ and ‘Both Aboriginal and Torres Strait Islander’ into a dummy variable that takes the value 1 if the individual comes from any Australian Indigenous background and the value 0 otherwise. This is our key explanatory variable of interest. We exclude individuals who are not born in Australia, as they are not of key interest and the diversity of their ancestry, language and cultural backgrounds would complicate the analyses unnecessarily.

It must be noted that the HILDA Survey is not fully nationally representative of the Australian population. Other than the typical exclusions of institutionalized individuals and the homeless, the HILDA Survey did not originally sample geographical areas within Australia that are classified as ‘very remote’ (although it did follow respondents who moved into them) [38]. While it is true that Indigenous Australians are overrepresented in ‘very remote’ areas, only a minority of the Indigenous population (around 15%) lives in them [40].

As can be observed in Table 1, in our data there are 3,815 (3%) observations from 814 individuals who self-identify as Indigenous, and 125,088 (97%) observations from 19,894 individuals who self-identify as non-Indigenous.

**Table 1:** Variable means for the whole sample and by Indigenous background.

	Means / Proportion of cases			Diff.
	All individuals	Non-Indigenous	Indigenous	
*Outcome variables*				
Somebody influenced responses	0.093	0.092	0.102	-0.010*
Suspicious after interview	0.018	0.018	0.024	-0.006**
Issues understanding questions	0.032	0.030	0.113	-0.083***
Lack of cooperation	0.016	0.015	0.041	-0.026***
*Control variables*				
Female	0.528	0.526	0.585	-0.059***
Age in years	42.327	42.574	34.232	8.342***
Partnered	0.595	0.600	0.427	0.173***
Number of adults in household	2.301	2.298	2.399	-0.101***
Number of children in household	0.619	0.607	1.028	-0.421***
Both parents Australian/NZ born	0.766	0.762	0.892	-0.130***
One parent overseas born	0.234	0.238	0.108	0.130***
Both parents overseas born	0.086	0.088	0.015	0.073***
Degree	0.187	0.191	0.072	0.119***
Certificate or diploma	0.278	0.279	0.233	0.046***
Year 12 education	0.151	0.151	0.147	0.004
Below year 12 education	0.384	0.379	0.548	-0.169***
Employed	0.654	0.660	0.468	0.192***
Not in the labour force	0.308	0.305	0.406	-0.101***
Unemployed	0.038	0.035	0.126	-0.091***
Income (in $10,000s)	8.138	8.197	6.201	1.996***
Health condition or disability	0.259	0.258	0.289	-0.031***
Major urban area	0.573	0.578	0.418	0.160***
Inner regional area	0.274	0.274	0.282	-0.008***
Outer regional, remote or very remote area	0.152	0.148	0.300	-0.152
Deprivation: 1st quintile	0.209	0.204	0.390	-0.186***
Deprivation: 2nd quintile	0.209	0.206	0.308	-0.102***
Deprivation: 3rd quintile	0.193	0.195	0.136	0.059***
Deprivation: 4th quintile	0.192	0.194	0.117	0.077***
Deprivation: 5th quintile	0.197	0.202	0.050	0.152***
New South Wales	0.297	0.298	0.253	0.045***
Victoria	0.246	0.250	0.111	0.139***
Queensland	0.217	0.215	0.301	-0.086***
Southern Australia	0.095	0.094	0.145	-0.051***
Western Australia	0.086	0.086	0.079	0.007
Tasmania	0.035	0.033	0.093	-0.060***
Northern Territory	0.006	0.006	0.003	0.003*
Australian Capital Territory	0.018	0.018	0.015	0.003
Reading or language problems	0.011	0.010	0.046	-0.036***
Times previously interviewed	5.207	5.226	4.565	0.661***
Interview length, in minutes	33.056	33.011	34.530	-1.519***
First contact with interviewer	0.519	0.516	0.597	-0.081***
Interviewer workload	95.790	95.713	98.328	-2.615***
Year of interview	2006.809	2007.311	2006.794	-0.516***
n (individuals)	20,708	19,894	814	
n (observations)	128,903	125,088	3,815	

Notes: HILDA Survey data (2001–2012). Significance levels on t-tests comparing group means:

^+^ p<0.1,

* p<0.05,

** p<0.01,

*** p<0.001.

The HILDA Survey contains rich information on the perceived quality of different aspects of the face-to-face interview, as reported by the interviewer right after its conclusion. We use this information to create four binary outcome variables that tap different dimensions of QotIP. The first outcome variable contains information on whether an adult person that was present in the room at the time of interview influenced the responses given by the respondent. The second outcome captures whether the interviewee seemed suspicious about the study after the interview. The third captures whether the respondent had issues understanding the questions posed by the interviewer. The fourth and final outcome variable identifies respondents who showed a lack of cooperation during the interview.

The first outcome variable (responses influenced) was constructed by coding response ‘Not at all’ with a value of 0, and responses ‘A little’, ‘A fair amount’ and ‘A great deal’ with a value of 1. The second outcome variable (respondent was suspicious) was constructed by coding response ‘No, not at all suspicious’ with a value of 0, and responses ‘Yes, somewhat suspicious’ and ‘Yes, very suspicious’ with a value of 1. The last two outcome variables (issues understanding questions and respondent cooperation) were constructed by coding responses ‘excellent’ and ‘very good’ with a value of 0 and responses ‘fair’, ‘poor’ and ‘very poor’ with a value of 1. Therefore, all 4 analytical outcome variables are dummy variables where a value of 1 indicates a suboptimal interview outcome, and a value of 0 indicates an optimal interview outcome. The advantages of collapsing categories in this manner are manifold. It helps to compare model coefficients across outcome variables, estimate more complex models where ordered variables are problematic, and correct for the fact that very few individuals fall into certain response categories. Sensitivity analyses using ordered response variables revealed no qualitative differences to the results.

Taken together, these four outcomes provide rich insights into overall QotIP. They are also complementary, as evidenced by fairly low pairwise correlations between them—from 0.04 (influenced and suspicious) to 0.26 (understanding and cooperation). It is nevertheless worth pointing out that all of these outcomes are subjective, as different interviewers might have different perceptions of what constitutes lack of cooperation or suspiciousness. To account for this, it is necessary to incorporate interviewer effects in multivariate models, as we will discuss later.

The distribution of QotIP proxy variables for the whole sample and by Indigenous status is shown at the top of Table 1. This is evidence that although suboptimal interview outcomes are rare in the HILDA Survey data (from 1.6% to 9.3% of all interviews depending on the outcome considered), they are more prevalent amongst Indigenous Australians. The responses of 10.2% of Indigenous Australians and 9.2% of non-Indigenous Australians appeared to be influenced by another person, while 2.4% of Indigenous Australians compared to 1.8% of non-Indigenous Australians were suspicious of the study. More strikingly, 11.3% of Indigenous Australians but just 3% of non-Indigenous Australians had issues understanding the survey questions, and 4.1% of Indigenous Australians but only 1.5% of other Australians were reportedly uncooperative. All of these discrepancies are statistically significant at conventional levels, which constitutes descriptive evidence of an ethnic effect on QotIP and is consistent with Hypothesis 1.

**Fig 1:**
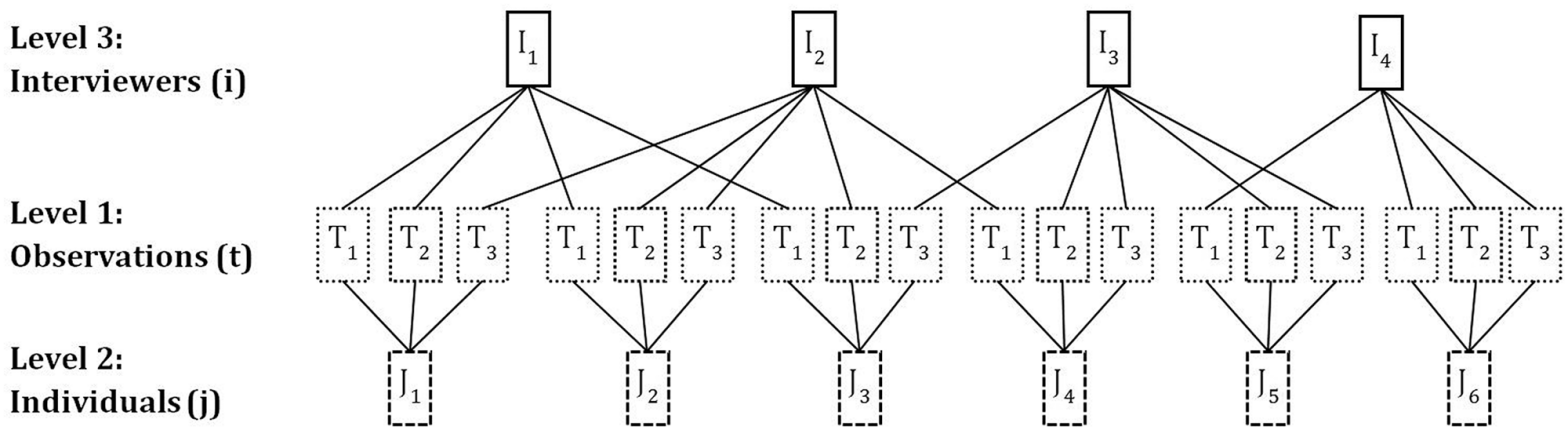
Example of multiple-membership three-level hierarchical data structure.

More robust and telling evidence can be gathered from multivariate models that control for observable and unobservable confounders and that take into account both the panel structure of the HILDA Survey data and any interviewer effects. To accommodate these complexities and fully exploit the properties of the data, we model the relationships between QotIP, Indigenous ethnic background and other relevant factors using multilevel models (i.e. hierarchical models) [41–43]. Specifically, we use three-level models where person-year observations are nested within survey respondents, who are in turn nested within interviewers. Therefore, the Level 1 units are person-year observations, the Level 2 units are survey respondents, and the Level 3 units are interviewers (see [44–46] for a similar set up). An added complexity of the data structure is that the same interviewer can interview many respondents (within and across survey waves) and the same survey respondent can be interviewed by different interviewers over the observation window, and so the nesting in the data is not ‘pure’ (see Fig 1 for an example). Therefore, our model must (and does) allow for multiple memberships [47–49].

Because our outcome variables of interest are dichotomous, we estimate the models using logistic regression. Let us denote a dichotomous variable of interest capturing a given interviewer observation as IO_ijt_, where individual i is interviewed by interviewer j at wave t. Our multiple-membership model takes the form:

$$\text{log}\left(\frac{{\text{Pr(IO}}_{\text{ijt}}\text{ = 1)}}{{\text{1-Pr(IO}}_{\text{ijt}}\text{ = 1)}}\right){\;=\;\beta }_{\text{0}}{\;+\beta }_{\text{1}}{\text{ IB}}_{\text{ij}}{\;+\beta }_{\text{2}}{\text{X}}_{\text{ijt}}\text{+}\overset{\text{T}}{\underset{\text{t}}{\sum }}{\text{w}}_{\text{ijt}}{\text{u}}_{\text{jt}}{\text{+v}}_{\text{ij}}{\text{+e}}_{\text{ijt}}$$ (1)

In this model, β_0_ the model’s grand intercept; IB denotes Indigenous background and β_1_ is its associated estimated coefficient; X is a vector of control variables and β_2_ is a transposed vector of their associated estimated coefficients; v_ij_ are the individual-level random effects capturing individual-specific unobserved heterogeneity; u_jt_ are the interviewer-level random effects capturing interviewer-specific unobserved heterogeneity; and e_ijt_ is the stochastic error term. In practice, in the multiple-membership model each respondent is assigned a single interviewer effect (u_j_) which is a weighted average of the effect for each of its different interviewers over time. For each individual, the weights add up to one (i.e.$\overset{\text{T}}{\underset{\text{t}}{\sum }}{\text{w}}_{\text{ijt}}=1$).

The X vector of control variables includes a wide array of variables known or suspected to affect the quality of the information gathered in the survey interview that might differ by ethnic background. These comprise variables capturing socio-demographic traits (gender, age and its square, partnership status, number of adults living in the household, number of children living in the household, second generation migrant status), measures of economic and human capital (highest educational qualification attained, employment status, annual household disposable income, presence of a long term condition, impairment or disability), geographical area characteristics (area of residence remoteness, area of residence socioeconomic deprivation, state of residence), and interview conditions (number of times the respondent was previously interviewed, whether the respondent had reading or language problems, interview workload, interview length and its square, and year of interview).

The models were estimated using MLwiN 2.25 software from within Stata 13 software, using the user-written subroutine 'runmlwin', and Markov Chain Monte Carlo (MCMC) methods [49].

## Results

Sample means on the analytical variables for the sample as a whole and for Indigenous and non-Indigenous ethnic groups separately are presented towards the bottom of Table 1. Of key interest here are the large and statistically significant ethnic divergences in most variables, with few exceptions (having year 12 as one’s highest educational credential, and living in an inner regional area, Western Australia or the Australian Capital Territory). Relative to Indigenous Australians, non-Indigenous Australians enjoy higher levels of human and economic capital, live in less populated households and in less deprived areas, have been interviewed more times, are less likely to experience a change in their interviewer and to display reading or language problems during the interview. This suggests that some of these factors might mediate the relationships between Indigenous Australian background and QotIP.

**Fig 2:**
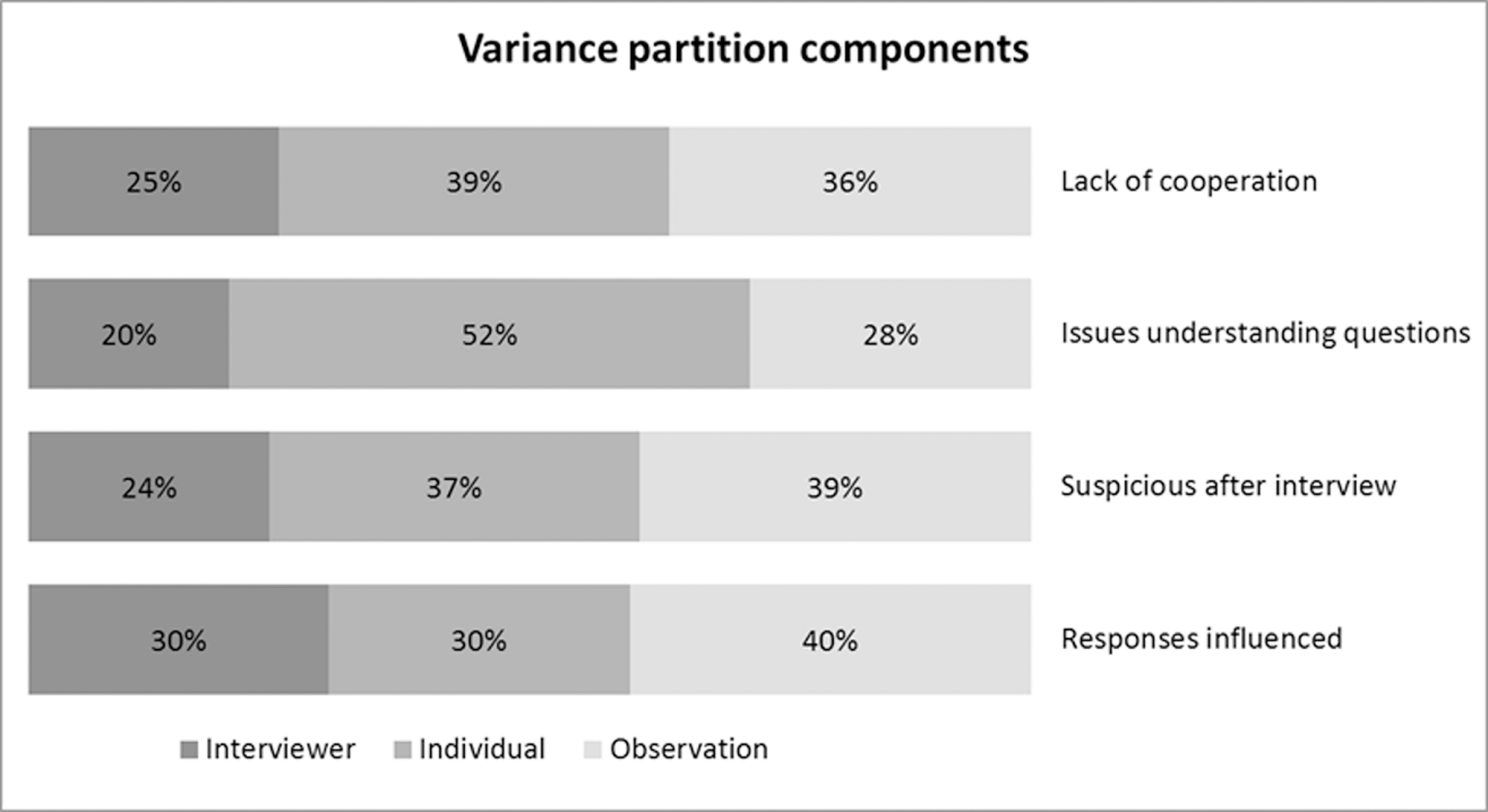
Variance partition components. HILDA Survey data (2001–2012). Multiple-membership three-level logistic regression models with no predictors. n (int) = 542; n (ind) = 20,708; n (obs) = 128,903.

We want to establish whether Indigenous ethnic background is associated with differential QotIP amongst the Australian population. To do so, we fit a series of multiple-membership three-level logistic regression models. As is typical when using multilevel specifications, we begin with a null model (i.e. a model with no explanatory variables) to determine the relative shares of the total variance in the outcome variables that can be attributed to interviewers, individuals and observations. This is achieved by calculating the variance partition components (VPCs) as follows:

$${\text{VPC}}_{\text{int}}\text{ = }\frac{\sigma_{\text{u}}^{2}\;}{\sigma_{\text{u}}^{2}{+\sigma }_{\text{v}}^{2}{+\sigma }_{\text{e}}^{2}}$$ (2)

$${\text{VPC}}_{\text{ind}}\text{ = }\frac{\sigma_{\text{v}}^{2}\;}{\sigma_{\text{u}}^{2}{+\sigma }_{\text{v}}^{2}{+\sigma }_{\text{e}}^{2}}$$ (3)

$${\text{VPC}}_{\text{obs}}\text{ = }\frac{\sigma_{\text{e}}^{2}\;}{\sigma_{\text{u}}^{2}{+\sigma }_{\text{v}}^{2}{+\sigma }_{\text{e}}^{2}}$$ (4)

where $\sigma_{\text{u}}^{2}$ represents the share of the variance that is between interviewers (i.e. interviewer-specific heterogeneity), $\sigma_{\text{v}}^{2}$ represents the share of the variance that is between individuals (i.e. person-specific heterogeneity), and $\sigma_{\text{e}}^{2}$ represents the share of the variance that is the observation-level residual variability. Note that because we use logistic regression models, the residual variance term $\sigma_{\text{e}}^{2}$ is $\frac{\pi^{\text{2}}}{\text{3}}\text{ = 3.29}$ ([42], page 110). Results are shown in Fig 2. The percentage of the total variance in our four outcome variables that is at the interviewer level is fairly similar, ranging from 20% (issues understanding questions) to 30% (responses influenced), and so are the analogous percentages for the observation level, ranging from 28% (issues understanding questions) to 40% (responses influenced). The percentages of the variance which is at the individual level are the most volatile, ranging from 30% (responses influenced) to 52% (issues understanding questions). Overall, the three variance components are substantial. Specifically, the large shares of the variance that are due to individual and interviewer effects evidence that using a model that incorporates levels for individuals and interviewers is necessary.

We now proceed to estimate models in which the only predictor variable is Indigenous ethnic background. These will further test Hypothesis 1 by probing for a ‘raw’ association between Indigenous background and QotIP net of individual and interviewer effects. For simplicity and as is common in logistic regression, we express the predicted effects of explanatory variables on the outcome variable using exponentiated coefficients, i.e. odds ratios. The estimated odds ratios from these models can be found in columns labelled ‘(i)’ in Table 2. Results indicate that there are statistically significant ethnic effects. Indigenous Australians have greater odds of having their responses influenced by a third person (odds ratio = 1.237, p<0.05), being suspicious after the interview (odds ratio = 1.652, p<0.01), having issues understanding the survey questions (odds ratio = 11.784, p<0.001), and being reportedly uncooperative during the interview (odds ratio = 4.243, p<0.001). The magnitudes of the last two effects are strikingly large. Altogether, these results provide strong evidence of an association between Indigenous background and all of the approximate measures of QotIP and are thus consistent with Hypothesis 1.

**Table 2:** Odds ratios from multiple-membership three-level logistic regression models

	Somebody Influenced responses		Suspicious after interview		Issues understanding questions		Lack of cooperation	
	(i)	(ii)	(i)	(ii)	(i)	(ii)	(i)	(ii)
*Indigeneity*								
ATSI	1.237*	0.932	1.652**	1.755**	11.784***	4.140***	4.243***	3.153***
*Socio-demographics*								
Female		0.756***		0.777***		0.746***		0.724***
Age		0.872***		1.113***		0.924***		1.047***
Age squared		1.001***		0.999***		1.001***		1.000***
Partnered		3.943***		0.843**		0.559***		0.602***
Number of adults in the household		1.259***		0.940(*)		1.180***		1.129***
Number of children in the household		1.089***		0.953		1.067**		0.989
Both parents Australian/NZ born *(ref*.*)*								
One parent overseas born		1.170**		0.933		1.032		0.835(*)
Both parents overseas born		0.808**		1.645***		0.809(*)		1.415*
*Human and economic capital*								
Degree *(ref*.*)*								
Certificate or diploma		1.214**		1.145		1.914***		1.321*
Year 12 education		0.903		1.234(*)		1.629***		1.328*
Below year 12 education		1.858***		1.220*		4.202***		1.508***
Employed *(ref*.*)*								
Not in the labour force		1.181***		0.931		1.334***		0.883
Unemployed		1.042		1.426**		1.930***		1.317*
Income (in $10,000s)		0.997		0.992		0.960***		0.992
Health condition or disability		1.361***		0.971		1.963***		1.298***
*Geographical area*								
Major urban area *(ref*.*)*								
Inner regional area		1.106(*)		0.851		1.069		1.113
Outer regional, remote or very remote area		1.157(*)		0.995		1.134		0.970
Deprivation: 1^st^ quintile *(ref*.*)*								
Deprivation: 2^nd^ quintile		1.024		1.215*		0.910		0.993
Deprivation: 3^rd^ quintile		0.957		0.970		0.767**		0.812*
Deprivation: 4^th^ quintile		1.039		1.230(*)		0.638***		0.968
Deprivation: 5^th^ quintile		0.861*		1.124		0.572***		0.865
New South Wales *(ref*.*)*								
Victoria		1.183		1.115		1.063		1.333
Queensland		1.054		0.765(*)		1.209		0.880
Southern Australia		1.885***		0.967		1.187		1.284
Western Australia		1.099		1.032		0.916		1.288
Tasmania		0.903		1.182		0.427*		0.421(*)
Northern Territory		0.704		0.705		1.439		0.947
Australian Capital Territory		1.087		0.363*		1.606		1.684
*Interview conditions*								
Reading or language problems		3.147***		1.522*		15.192***		3.261***
Times previously interviewed		0.942***		0.852***		0.950***		0.953***
Interview length		1.006**		0.995		1.016**		0.972**
Interview length squared		1.000(*)		1.000(*)		1.000		1.000**
First contact with interviewer		1.188***		1.612***		1.305***		1.525***
Interviewer workload		0.999		1.003**		1.000		1.001
Year of interview		0.996***		0.938***		1.000*		0.979***
n (interviewers)	542							
n (individuals)	20,708							
n (observations)	128,903							
Chains	5,000							
Burns	500							
% variance at interviewer level	24%	24%	30%	31%	20%	26%	26%	26%
% variance at individual level	37%	35%	30%	24%	51%	33%	39%	36%
% variance at observation level	39%	41%	41%	45%	30%	41%	35%	38%
DIC	58,549	56,542	17,540	17,056	24,180	23,281	15,490	15,451

Notes: HILDA Survey data (2001–2012). Significance levels on odds ratios:

^+^ p<0.1,

* p<0.05,

** p<0.01,

*** p<0.001.

Testing counter Hypotheses 2a and 2b requires fitting our full and preferred specifications in which all the observable explanatory variables of interest are added to the model. If the odds ratios on the Indigenous status variable remain statistically significant, then we would conclude that an independent effect exists (Hypothesis 2a). If the statistical significance of the odds ratios on the Indigenous status variable fades, we would then conclude that the previously observed association was indirect and mediated by the observable explanatory variables added to the models (Hypothesis 2b). The results from our fully specified models are presented in columns labelled ‘(ii)’ in Table 2. In these specifications, there is no longer a statistically significant effect of Indigenous background on the odds of respondents having their responses influenced (odds ratio = 0.932, p>0.1). However, the odds of a respondent being suspicious of the study after the interview are 75.5% larger when the respondent is Indigenous than when the respondent is not Indigenous (odds ratio = 1.755, p<0.01). More strikingly, the odds of having issues understanding the survey question multiply more than fourfold with Indigeneity (odds ratio = 4.140, p<0.001), and the odds of the respondent being reportedly uncooperative during the interview multiply more than threefold (odds ratio = 3.153, p<0.001). Therefore, our results are consistent with the hypothesis that there are independent effects of Indigeneity on three of the approximate measures of QotIP (being suspicious of the study, having issues understanding survey questions and being uncooperative during the interview), and indirect effects for one of them (responses being influenced by another person).

Though not the focus of this paper, our models offer other interesting insights. Having a degree as one’s highest educational qualification (relative to any other qualification), being employed (relative to being unemployed), having no reading or language problems during the interview, being female, having been previously interviewed a higher number of times, and being interviewed by a known interviewer are all associated with higher QotIP. There is more mixed evidence for explanatory variables capturing age and its square, being partnered, being out of the labour force (rather than employed), household income, the number of adults in the household, the number of children in the household, having a lasting health condition, the length of the interview and its square, interviewer workload, and year of interview. The effects on the outcomes of variables capturing area-level characteristics and region of residence are less pronounced. The positive effects observed for second generation migrants (‘Both parents overseas born’ variable) on cooperation (odds ratio = 1.415, p<0.05) and suspicion (odds ratio = 1.645, p<0.001) are particularly interesting. Similar to Indigenous Australians, people with two parents born overseas are more likely to be suspicious and uncooperative during interviews net of socio-economic status and unobserved sources of heterogeneity, which hints at the interplay of cultural processes and the survey protocols.

Our key results and their implications will be discussed in more depth in the concluding section that follows.

## Conclusion and Discussion

In this paper we have used rich data from the Household, Income and Labour Dynamics in Australia Survey to estimate multiple-membership three-level regression models on a number of approximate measures of the quality of the interview process. In doing so, we have gathered robust and consistent evidence of divergences in the quality of the interview process between Indigenous and non-Indigenous Australians when faced with a survey vehicle designed primary for enumeration of the general population. Indigenous people are significantly (and often substantially) more likely than non-Indigenous people in Australia to have issues understanding survey questions, be suspicious of the study, be uncooperative, and have their responses influenced by another person. All but the latter appear to be independent effects of Indigeneity, rather than channeled by observable socio-economic, socio-demographic and area-level characteristics unevenly distributed amongst Indigenous and non-Indigenous Australians. It is worth restating that the HILDA Survey did not originally sample remote Indigenous communities (though it follows respondents who moved there). Since traditional Indigenous culture is stronger and more widespread in remote locations [28], then the observed effects are likely to be lower bound estimates of the true effects of Indigenous culture on QotIP. To the extent that QotIP is predictive of survey data quality, the information gathered from Indigenous Australians using general population surveys is likely to be of poorer quality than that gathered from non-Indigenous Australians. These findings add to the existing body of knowledge highlighting difficulties in involving and retaining Indigenous Australians in population surveys.

The fact that unexplained ethnic gaps in QotIP are observed for three related but distinct proxies of this concept reinforces our thesis that cultural effects are indeed at play. Some hypotheses about the cultural mechanisms driving these associations can be developed from early research on the inclusion of Indigenous Australians in mainstream social surveys, as well as from a cognate body of knowledge examining barriers and facilitators of culturally appropriate interactions between Indigenous Australians and Australian official institutions (particularly, the health system).

Relatively poor understanding of research questions net of English language proficiency amongst Indigenous Australians might be a product of ethnic differences in conversational practices and schemas. Ware reviews research on the ways in which miscommunication between Indigenous Australians and health service professionals emerge due to mismatches in ‘cultural models of what constitutes polite or constructive communication’ [50]. These include Indigenous Australians’ predisposition to say ‘yes’ or ‘no’ by default or staying silent when they do not fully understand a question or explanation due to embarrassment, to say ‘yes’ when they mean ‘no’, to respond to questions based on what they perceive the interlocutor may expect to hear—often assuming that the first of a range of options given to them is the preferred one, and to experience shame when being ‘put on the spotlight’ through a direct or sensitive question [20, 50–51]. Reservations amongst some Indigenous Australians to disclose personal information to members of the other sex or other ethnic or kinship groups and a dislike of ‘closed questions’ have also been documented [21, 50–51].

More worryingly perhaps are the ethnic divergences in respondent cooperation and suspicion, which might have roots in remaining ethnic tensions within contemporary Australian society. Intergenerational trauma due to colonization and forced separation amongst Indigenous Australians has been shown to have contemporary effects on life domains such as mental health [51–52]. There is also a body of evidence suggesting that this still influences Indigenous Australians’ interactions with official institutions and non-Indigenous Australians [50, 53–54]. Research has found that Indigenous Australians experience anxiety when confronted with unfamiliar situations involving non-Indigenous public officials, fear due to historical as well as more recent removals of children from their families, mistrust due to ongoing individual, systemic and institutional racism, and shame and shyness due to previous negative interactions with non-Indigenous authorities or perceived social distance with their interlocutor [50]. Many of these elements are present in traditional survey interviews. Behaviors such as maintaining eye contact, sitting in close proximity, or using first names in conversation with strangers, which are also common in an interview setting, go against Indigenous cultural protocols too [50]. It has also been argued that the presence of a researcher or interviewer to ask questions in Indigenous homes can be perceived as an ‘intrusion’ because of the collective folk memory on past and present treatment of Indigenous Australians [20]. If our findings indicate that Indigenous Australians are less comfortable during interviews, there might be associated ethical issues about using culturally unadapted survey protocols for this subpopulation.

However, these explanations, while considered in the extant research, are by no means definitive, as our analyses cannot elucidate the specific mechanisms linking Indigeneity and QotIP. Future research might be able to address this in several ways. First, new quantitative studies would benefit from specifically-tailored datasets collecting information on the potential mechanisms leading to poor QotIP, such as those suggested above. Second, qualitative studies could also shed light over the mechanisms linking Indigeneity and QotIP. For example, in-depth interviews or focus groups about the experience of being interviewed could be conducted with subsets of (Indigenous and non-Indigenous) survey participants for whom good and poor QotIP was reported. Third, content analysis of interviewer-respondent interactions could be undertaken using *verbatim* transcripts of the survey interview, as a way to determine the sources of conversational misunderstandings [55].

These findings have important practical implications for the validity of studies based on general population surveys that incorporate Indigenous individuals. Any analyses of the Indigenous subsample might produce unreliable results due to differences in interpretation and general survey engagement between Indigenous and non-Indigenous respondents. This problem is particularly acute for comparative analyses of Indigenous and non-Indigenous respondents, but to a certain extent also applies to estimates pertaining general samples—particularly when the (sub)population of interest incorporates a large share of Indigenous respondents. This poses serious challenges in using survey data to inform the design of evidence-based policy levers aimed at ‘closing the gaps’ between Indigenous and non-Indigenous Australians and monitor their progress.

Our results are thus indicative that asking questions in the same way to everyone may not be the best way to obtain the ‘right’ answer in the Australian context, given ethnic-based cultural heterogeneity. In particular, any instruments designed to collect data from Indigenous Australians should be designed as to ensure that the information collected is culturally relevant and collection methods are sensitive to Indigenous culture and normative expectations. We see two main routes through which more valid and comparable data on Indigenous Australians could be collected.

First, new social surveys could feature separate components for Indigenous and non-Indigenous Australians, with survey protocols that match the needs and cultural practices of each of these subpopulations. Some recent Australian surveys that have been specifically tailored to collect information from Indigenous Australians include the National Aboriginal and Torres Strait Islander Health Survey (NATSIHS), the Longitudinal Study of Indigenous Children (LSIC), and the National Aboriginal and Torres Strait Islander Social Survey (NATSISS). These were preceded by intense community consultation with Indigenous community leaders and questionnaire testing with Indigenous populations and, as a result, accommodate Indigenous ways of understanding questions and response categories and ensure appropriate alignment between respondent and researcher interpretation of survey items.

However, most population surveys in Australia, including the HILDA Survey and LSAC are not designed to specifically accommodate Indigenous cultural and interpretative needs, but instead focus on promoting measurement fidelity, flow and question interpretability among the sample majority. In the light of our findings, if these surveys are to be used credibly to understand Indigenous outcomes they should move away from rigid ‘ethnocentric’ data collection instruments and delivery modes and enhance their flexibility to incorporate Indigenous Australian cultural norms. This course of action may involve the development and implementation of survey sub-protocols for Indigenous sample members that are sensitive to Indigenous cultural perspectives. More research is needed to determine the specific ways in which this could be achieved.

One potential course of action is to use interviewer-respondent ethnic matching by employing and training Indigenous interviewers to work in geographical areas where Indigenous Australians are overrepresented [11, 56]. In Australia, previous attempts to accomplish this in the 1990s were largely unsuccessful because of difficulties finding suitably qualified Indigenous Australians [20]. Yet, the number of Indigenous Australians with the necessary skillset to undertake survey interviewing is likely much greater now than then—as reflected, for example, by increases in the number of enrolments of Indigenous Australians in tertiary education [57]. However, there are still inherent complexities in successfully implementing this method. For example, Indigenous Australians may be reluctant to reveal information to Indigenous people of a different gender or from different family, language, kinship or ethnic groups [50–51]. They may also be hesitant to share culturally-sensitive information with Indigenous people of their same group, given Indigenous collective models of information ownership and the potentially devastating consequences for the respondent of disclosure of culturally-inappropriate responses and behaviors [20, 51, 53]. Another potential course of action is to train all, Indigenous and non-Indigenous, survey interviewers in ‘cultural competence skills’, in line with current directions on the interactions between Indigenous Australians and the Australian health system [50, 51]. Survey questions may not need to be changed to get good quality data from Indigenous respondents, but the interview situation should be culturally appropriate.

Despite the innovative nature and important contributions of this study, certain limitations need to be acknowledged. These are also suggestive of areas for further inquiry that may prove fruitful. First, due to data limitations, our analyses are not sensitive to socio-economic and cultural heterogeneity between different Indigenous populations across Australia. Second, we argue that any effects of Indigeneity on survey quality that remain after controlling for a wide range of socio-economic predictors and unobserved sources of heterogeneity are likely attributable to culture, i.e. mismatches between Indigenous cultural protocols and survey interview protocols. Yet, this is only a *proxy* for culture. Recent quantitative research on Indigenous Australians in other fields has attempted to measure culture more directly by considering adherence to Indigenous protocols, norms and traditions and participation in Indigenous rituals and social activities [58]. This requires very specific data, such as those available in NATSISS. Future research might extend our findings using more direct measures of Indigenous culture. Third, data availability restrictions did not permit us to incorporate some relevant interviewer characteristics, such as gender and experience, into the analyses. Using such information has the potential to shed light over whether the observed ‘gap’ in outcomes disfavoring Indigenous respondents reduces when their interviewers have certain traits, for instance, when the interviewer is of Indigenous descent [20]. Also, we know little about the interviewer-respondent matching practices used by the agency responsible for collecting the HILDA Survey data. While it is possible that non-random matching practices correlated with respondents’ ethnicity may introduce some bias to our results (see [46]), accounting for interviewer effects is the most powerful available way to minimize any distortions. Altogether, these data shortcomings mean that it is not possible to fully rule out interviewer xenophobia as a plausible explanation for the Indigeneity effect.

While our focus is on the Indigenous people of Australia, our results are likely to extend to other collectives and contexts. First, it is likely that the findings reported here apply to other Indigenous peoples across the world. This is because, as Indigenous Australians, the Indigenous populations in countries such as New Zealand, Canada, United States, or Taiwan—to name a few—tend to experience high levels of economic disadvantage and social exclusion, have a different mother-tongue language than members of the ethnic majority, and have distinctive cultural norms and practices [59–63]. However, it must be noted that the socio-economic gaps relative to the ethnic majority experienced by Indigenous Australians are more pronounced than those between Indigenous and non-Indigenous populations in other first-world nations [59–60, 64]. Second, Indigeneity is only one of many potentially marginalized statuses within contemporary Australian society [19]. Based on our results, it is highly likely that other dimensions of social exclusion also hinder QotIP. It is possible that other marginalized statuses not only have independent effects of their own, but also interact cumulatively with each other and with Indigenous background. In both cases, our methodological approach would be useful to inform subsequent research on these issues. We have paved the way for this to be accomplished, and more studies devoted to systematically exploring these issues would advance the field.
